# Effectiveness of mouthwashes on reducing SARS-CoV-2 viral load in oral cavity: a systematic review and meta-analysis

**DOI:** 10.1186/s12903-023-03126-4

**Published:** 2023-07-03

**Authors:** Tayebe Ebrahimi, Ahmad Reza Shamshiri, Masoud Alebouyeh, Simin Z. Mohebbi

**Affiliations:** 1 Research Center for Caries Prevention, Dentistry Research Institute, Tehran University of Medical Sciences, Tehran, Iran; 2 Community Oral Health Department, School of Dentistry, Tehran University of Medical Sciences, Tehran, Iran; 3 Pediatric Infections Research Centre, Research Institute for Children’s Health, Shahid Beheshti University of Medical Sciences, Tehran, Iran

**Keywords:** Mouthwash, SARS-CoV-2, Viral load

## Abstract

**Background:**

The risk of SARS-COV-2 transmission is relatively high during dental procedures. A study was conducted to investigate the effects of mouthwashes on SARS-COV-2 viral load reduction in the oral cavity.

**Methods:**

A systematic search was performed in PubMed, EMBASE, Scopus, Web of Science, and Cochrane library for relevant studies up to 20 July, 2022. Randomized and non-randomized clinical trial and quasi-experimental studies evaluating patients with Covid-19 infection (patients) who used mouthwashes (intervention) compared to the same patients before using the mouthwash (comparison) for reducing the SARS-COV-2 load or increasing the cycle threshold (Ct) value (outcome) were searched according to PICO components. Three independent reviewers conducted literature screening and data extraction. The Modified Downs and Black checklist was used for quality assessment. A meta-analysis was performed with a random effects model in the Revman 5.4.1software using the mean difference (MD) of cycle threshold (Ct) values.

**Results:**

Of 1653 articles, 9 with a high methodological quality were included. A meta-analysis indicated that 1% Povidone-iodine (PVP-I) was an effective mouthwash for reducing the SARS-COV-2 viral load [MD 3.61 (95% confidence interval 1.03, 6.19)]. Cetylpyridinium chloride (CPC) [MD 0.61 (95% confidence interval -1.03, 2.25)] and Chlorhexidine gluconate (CHX) [MD -0.04 95% confidence interval (-1.20, 1.12)] were not effective against SARS-COV-2.

**Conclusion:**

Using mouthwashes containing PVP-I may be recommended for reducing the SARS-COV-2 viral load in the oral cavity of patients before and during dental procedures, while the evidence is not sufficient for such effects for CPC and CHX-containing mouthwashes.

## Introduction

SARS-CoV-2, the cause of coronavirus disease 2019 (Covid-19), a Betacoronavirus, belongs to the coronaviride family. It is a single-stranded, positive-sense RNA virus [1]. The main transmission route of SARS-CoV-2 is through respiratory droplets. These droplets cause direct contact infection during coughing, sneezing, and speaking or indirect contact infection via touching infected objects and the environment [2]. This virus shows high transmissibility and binds with the surface angiotensin-converting enzyme-2 (ACE2) receptors of host cells using the S1 subunit of the receptor binding domain in the spike protein. These receptors are expressed in multiple human systems and tissues, such as the lung and salivary glands as well as the epithelial cells of the nasopharynx and oropharynx [1, 3–5].

There is evidence that the oral cavity is a SARS-CoV-2 reservoir because ACE2 is highly expressed in the oral non-keratinizing squamous epithelium. Moreover, researchers successfully detected the SARS-CoV-2 RNA in the saliva [6]. Therefore, saliva is a source of SARS-CoV-2 transmission. There is a relatively high risk of virus transmission in dental procedures and oropharyngeal examination because of face-to-face treatments and aerosol-generating equipment [7, 8]. The ultrasonic scalers and high-speed handpieces spray saliva, blood, and fomites resulting in microbial transmission between patients and clinic staff. Viral shedding has been detected in the oral cavity of symptomatic and asymptomatic patients [9].

Prevention of SARS-CoV-2 infection is important in dental clinics; hence, it is critical to break the viral transmission chain between the patients and staff. There are some recommendations for this. The first step is to use personal protective equipment. Patient evaluation and identification of patients with potential Covid-19 infection are very crucial. The use of a non-contact thermometer is recommended for temperature measurement. A questionnaire can screen the patients; it should investigate whether the patient had any Covid-19 infection symptoms, such as fever and respiratory problems, during the past 14 days and if they had a close contact with a confirmed Covid-19 infected patient within the past two weeks [10]. Moreover, postponing the appointment and referring the patients to local health departments is recommended if the patient has a body temperature above 37.3 °C or is suspected as an at-risk case with a positive answer to the Covid-19 infection questionnaire [11].

Despite the application of these health recommendations, because of the presence of asymptomatic patients in to dental clinics, additional protective measures should be considered before and during dental procedures, such as the use of disinfectants and mouthwashes.

Today, a large number of antimicrobial mouthwashes are available on the market that have natural or synthetic antiseptic compounds. Preoperative antisepsis mouthwashes are frequently used in dental offices [12]. Different concentrations of these mouthwashes have antibacterial and antiviral effects [13, 14].

Recent publications have recommended that using antiseptic mouthwashes may control the viral load of SARS-COV-2 in the saliva. However, scientific evidence is lacking/contradictory for the anti-SARS-COV-2 effects. Although researchers have investigated the in-vitro effects of antiseptic mouthwashes on Covid-19 [15–19], limited clinical trial studies have examined the effects of antiseptic mouthwashes on Covid-19 viral load. The present systematic review was performed to answer: What are the effects of mouthwashes on SARS-COV-2 viral load reduction in the oral cavity?

Methods.

We systematically reviewed studies including patients with a SARS-CoV-2 positive test that used a mouthwash for SARS-COV-2 viral load reduction. In this study, we adhered to the Preferred Reporting Items for Systematic Reviews and Meta-Analyses (PRISMA) statement 2020 recommendations provided by Liberati [20].

### Electronic searches

The PubMed, EMBASE, Scopus, Web of Science and Cochrane Central databases were searched using the MeSH and non-MeSH terms and the keywords. Table 1 presents the search strategy for mentioned databases. Google Scholar, MedRxiv, and clinicaltrials.gov were also searched with similar keywords manually to retrieve the gray literature. The reference lists of the included papers were also searched to find relevant studies.

**Table 1:** Search strategy in the searched databases according to PICO components

Pubmed	(“COVID 19” OR COVID19 OR COVID-19 OR “COVID-19 Virus” OR “COVID 19 Virus” OR “COVID-19 Viruses” OR (Virus AND COVID-19) OR “Wuhan Coronavirus” OR (Coronavirus AND Wuhan) OR “COVID19 Virus” OR “COVID19 Viruses” OR (Virus AND COVID19) OR (Viruses AND COVID19) OR “SARS-CoV-2 Infection” OR “SARS-CoV-2InfeCtions” OR (Infection AND SARS-CoV-2) OR “2019 Novel Coronavirus Disease” OR “2019 Novel Coronavirus Infection” OR “2019-nCoV Disease” OR “2019 nCoV Disease” OR “2019-nCoV Diseases” OR (Disease AND 2019-nCoV) OR “COVID-19 Virus Infection” OR “COVID 19 Virus Infection” OR “COVID-19 Virus Infections” OR (Infection AND “COVID-19 Virus”) OR “Coronavirus Disease 2019” OR (“Disease 2019” AND Coronavirus) OR “Coronavirus Disease-19” OR “Coronavirus Disease 19” OR “Severe Acute Respiratory Syndrome Coronavirus 2” OR “SARS Coronavirus 2 Infection” OR “COVID-19 Virus Disease” OR “COVID 19 Virus Disease” OR “COVID-19 Virus Diseases” OR (Disease AND “COVID-19 Virus”) OR (“Virus Disease” AND COVID-19) OR “2019-nCoV Infection” OR “2019 nCoV Infection” OR “2019-nCoV Infections” OR (Infection AND 2019-nCoV) AND “Mouth Rinse” OR “Mouth Rinses” OR “Mouth Bath” OR “Mouth Baths” OR “Mouth Wash”)
scopus	(ALL(“COVID 19”) OR ALL(COVID19) OR ALL(COVID-19) OR ALL(“COVID-19 Virus”) OR ALL(“COVID 19 Virus”) OR ALL(“COVID-19 Viruses”) OR ALL((Virus AND COVID-19)) OR ALL(“Wuhan Coronavirus”) OR ALL((Coronavirus AND Wuhan)) OR ALL(“COVID19 Virus”) OR ALL(“COVID19 Viruses”) OR ALL((Virus AND COVID19)) OR ALL((Viruses AND COVID19)) OR ALL(“SARS-CoV-2 Infection”) OR ALL(“SARS-CoV-2 Infections”) OR ALL((Infection AND SARS-CoV-2)) OR ALL(“2019 Novel Coronavirus Disease”) OR ALL(“2019 Novel Coronavirus Infection”) OR ALL(“2019-nCoV Disease”) OR ALL(“2019 nCoV Disease”) OR ALL(“2019-nCoV Diseases”) OR ALL((Disease AND 2019-nCoV)) OR ALL(“COVID-19 Virus Infection”) OR ALL(“COVID 19 Virus Infection”) OR ALL(“COVID-19 Virus Infections”) OR ALL((Infection AND “COVID-19 Virus”)) OR ALL(“Coronavirus Disease 2019”) OR ALL((“Disease 2019” AND Coronavirus)) OR ALL(“Coronavirus Disease-19”) OR ALL(“Coronavirus Disease 19”) OR ALL(“Severe Acute Respiratory Syndrome Coronavirus 2”) OR ALL(“SARS Coronavirus 2 Infection”) OR ALL(“COVID-19 Virus Disease”) OR ALL(“COVID 19 Virus Disease”) OR ALL(“COVID-19 Virus Diseases”) OR ALL((Disease AND “COVID-19 Virus”)) OR ((“Virus Disease” AND COVID-19)) OR ALL(“2019-nCoV Infection”) OR ALL(“2019 nCoV Infection”) OR ALL(“2019-nCoV Infections”) OR ALL((Infection AND 2019-nCoV))) AND (ALL(“Mouth Rinse”) OR ALL(“Mouth Rinses”) OR ALL(“Mouth Bath”) OR ALL(“Mouth Baths”) OR ALL(“Mouth Wash”)))
Embase	(“coronavirus infections” OR “coronavirus” OR “covid 2019” OR “SARS2” OR “SARS-CoV-2” OR “SARSCoV-19” OR “severe acute respiratory syndrome coronavirus 2” OR “coronavirus infection” OR “severe acute respiratory pneumonia outbreak” OR “novel cov” OR “2019ncov” OR “sars cov2” OR “cov2” OR “ncov” OR “covid-19” OR “covid19” OR “coronaviridae” OR “corona virus” OR “COVID-19 pandemic” OR “2019 novel coronavirus disease” OR “SARS-CoV-2 infection” OR “COVID-19 virus disease” OR “2019 novel coronavirus infection” OR “2019-nCoV infection” OR “coronavirus disease 2019” OR “coronavirus disease-19” OR “2019- nCoV disease” OR “COVID-19 virus infection” OR “2019-nCoV” OR “SARS-CoV-2”) AND (“mouthwashes” OR “Mouth Rinse” OR “Mouth Rinses” OR “Mouth Bath” OR “Mouth Baths” OR “mouthwash” OR “Mouth Wash”)
Web of science	(ALL = “COVID 19” OR ALL = COVID19 OR ALL = COVID-19 OR ALL = “COVID-19 Virus” ALL = “COVID 19 Virus” OR ALL = “COVID-19 Viruses” OR ALL = (Virus AND COVID-19) OR ALL = “Wuhan Coronavirus” OR ALL = (Coronavirus AND Wuhan) OR ALL = “COVID19 Virus” OR ALL = “COVID19 Viruses” OR ALL = (Virus AND COVID19) OR ALL = (Viruses AND COVID19) OR ALL = “SARS-CoV-2 Infection” OR ALL = “SARS-CoV-2 Infections” OR ALL = (Infection AND SARS-CoV-2) OR ALL = “2019 Novel Coronavirus Disease” OR ALL = “2019 Novel Coronavirus Infection” OR ALL = “2019-nCoV Disease” OR ALL = “2019 nCoV Disease” OR ALL = “2019-nCoV Diseases” OR ALL = (Disease AND 2019-nCoV) OR ALL = “COVID-19 Virus Infection” OR ALL = “COVID 19 Virus Infection” OR ALL = “COVID-19 Virus Infections” OR ALL = (Infection AND “COVID-19 Virus”) OR ALL = “Coronavirus Disease 2019” OR ALL = (“Disease 2019” AND Coronavirus) OR ALL = “Coronavirus Disease-19” OR ALL = “Coronavirus Disease 19” OR ALL = “Severe Acute Respiratory Syndrome Coronavirus 2” OR ALL = “SARS Coronavirus 2 Infection” OR ALL = “COVID-19 Virus Disease” OR ALL = “COVID 19 Virus Disease” OR ALL = “COVID-19 Virus Diseases” OR ALL = (Disease AND “COVID-19 Virus”) OR ALL = (“Virus Disease” AND COVID-19) OR ALL = “2019-nCoV Infection” OR ALL = “2019 nCoV Infection” OR ALL = “2019-nCoV Infections” OR ALL = (Infection AND 2019-nCoV)) AND (ALL = “Mouth Rinse” OR ALL = “Mouth Rinses” OR ALL = “Mouth Bath” OR ALL = “Mouth Baths” OR ALL = “Mouth Wash”))
Cochrane library	TI = (“coronavirus infections” OR “coronavirus” OR “covid 2019” OR “SARS2” OR “SARS-CoV-2” OR “SARSCoV-19” OR “severe acute respiratory syndrome coronavirus 2” OR “coronavirus infection” OR “severe acute respiratory pneumonia outbreak” OR “novel cov” OR “2019ncov” OR “sars cov2” OR “cov2” OR “ncov” OR “covid-19” OR “covid19” OR “coronaviridae” OR “corona virus” OR “COVID-19 pandemic” OR “2019 novel coronavirus disease” OR “SARS-CoV-2 infection” OR “COVID-19 virus disease” OR “2019 novel coronavirus infection” OR “coronavirus disease 2019” OR “coronavirus disease-19” OR “COVID-19 virus infection” OR “SARS-CoV-2” OR “2019-ncov infection” OR “2019-ncov disease” OR “2019-ncov” OR “sars-cov” OR “middle east respiratory syndrome coronavirus”OR “severe

### Eligibility criteria and study selection

The studies that fulfilled the following inclusion criteria according to the PICO acronym were included.

### Type of included studies

Randomized clinical trialsnon-randomized clinical trialsquasi-experimental studies

Types of participants: Participants were subjects diagnosed with Covid-19 infection with no age or gender restrictions.

### Types of interventions:

- Interventions: The use of the mouthwash was an intervention for patients infected with Covid-19
- Comparator: No mouthwash use was the comparison

### Types of outcome measures:

- Primary outcome: change in cycle threshold value.
- Secondary outcome: change in viral load.

### Types of excluded studies:

- Reviews
- Letters to the editor
- Technical notes
- In vitro studies
- Animal studies
- conference papers
- studies without the evaluation of the SARS-COV2 viral load or Ct values in saliva

### Data extraction

**Fig. 1:**
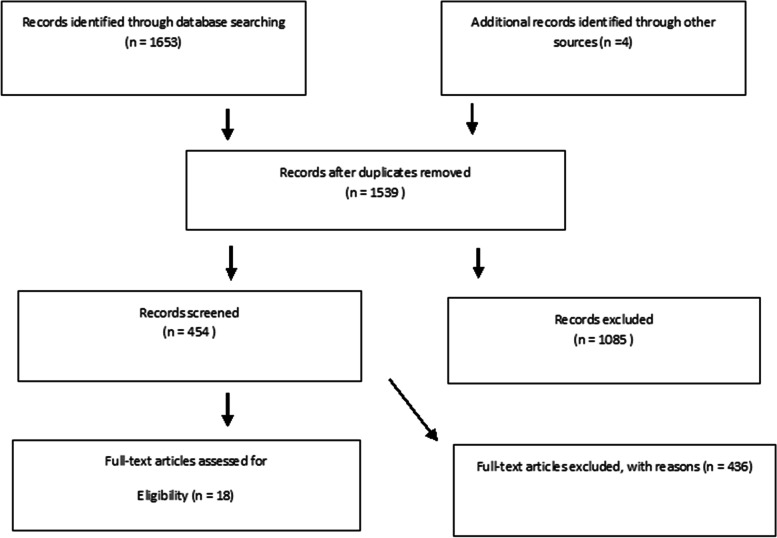
The PRISMA flow diagram of screening and selection process

The screening was done independently by T.E, SZ.M, and ARSH. The PRISMA flow diagram was used as a guide to the selection process (Fig. 1). First, duplicate results were identified and excluded. The titles and the abstracts of the papers were screened to exclude the irrelevant studies. Accordingly, the search results were categorized into three categories (included, excluded and unclear). Then, the full texts of the retrieved studies were reviewed for final inclusion. Any disagreement between the three researchers was resolved by discussion. The following data were extracted from eligible articles: study characteristics (study title, authors, date of publication, study design, number of patients); baseline data (kind of mouthwash, type of examination for measuring the viral load, type of analyses of viral load) and clinical outcomes (viral load reduction). The mean and standard deviation of Ct values or mean and standard deviation of viral load before and after the intervention were compared. This review study was conducted from November 2, 2020 to August 15, 2022. The Endnote 20 software was used for organizing the references.

### Assessing the risk of bias

Three reviewers (T.E, S.Z.M, A.SH) independently assessed the risk of bias for the included studies as part of the data extraction procedure. A modified Down and Black (D&B) Risk of Bias checklist [21] was used for assessing the quality of the included studies. Each satisfactory response received a score of 1; otherwise, a score of 0 was assigned. Studies with a modified D&B level ≥ 5 were considered as studies with a low risk of bias. Those with a modified D&B level < 5 points were considered as studies with a high risk of bias (Table 2). GRADE (Grades of Recommendation, Assessment, Development and Evaluation) system was applied to rank the certainty of the scientific evidence [22].

**Table 2:** The results of Modified Downs and Black checklist* for quality assessment

	Gottsaunerr et al. [23]	Mohamed et al. [24]	Mukhtar et al. [25]	Lamas et al. [26]	Yoon et al. [27]	Seneviratne et al. [28]	Carroul et al. [29]	Eduardo et al. [30]	Chaudhary et al. [31]
Objective Clearly Stated 1	1	1	1	1	1	1	1	1	1
Main outcomes clearly described ^2^	1	1	1	1	1	1	1	1	1
Patients characteristics clearly defined ^3^	1	1	1	1	1	1	1	1	1
Main findings clearly defined ^4^	1	1	1	1	1	1	1	1	0
Random variability in estimates provided ^5^	1	1	1	1	1	1	1	1	1
Sample targeted representative of population ^6^	0	0	1	0	0	0	1	1	1
Sample recruited representative of population ^7^	0	0	1	0	0	0	1	1	1
Primary outcomes valid/reliable ^8^	1	1	1	1	1	1	1	1	1
Total	6	6	8	6	6	6	8	8	7

^*^We obtained questions number 1,2,3,6, 7,11,12,20 of the D& B checklist

### Meta-analysis

Five studies that reported the mean and standard deviation of the Ct value or the value could be calculated from other reported data in the study were included in the meta-analysis. The RevMan 5.4.1 was used for analysis. There was a high level of heterogeneity in the mouthwash type, diagnostic kit, specimen (saliva or nasopharynx or oropharynx swab) and time of experiment (the time between the first RT-PCR test and using the mouthwash) among studies. Random-effects models and subgroup analysis were used to reduce the impact of heterogeneity. The Egger’s and Begg’s tests were used for publication bias assessment.

### Ethical consideration

This systematic review and meta-analysis was registered in the PROSPERO database (registration number: CRD42021274832).

## Results

In the initial search, 1653 papers were retrieved from the PubMed, EMBASE, Scopus, Web of Science, Cochrane Central, Google Scholar, MedRxiv, and clinicaltrials.gov. After removal of duplicates, 1539 title and abstracts were screened for the eligibility criteria. As for the remaining 18 articles, a paper was excluded if it met other inclusion criteria but did not report the mean and SD of the viral load or an accurate Ct value before and after the intervention. Excluded studies and reasons for exclusion are listed in Table 3. Finally, 9 articles were included in our study (See Fig. 1).

**Table 3:** Excluded studies and reasons for exclusion

Cyril et al. (2021) [32]	Viral load or Ct value changes was not reported
Anderson et al. (2022) [33]	In vitro study
Takeda et al. (2022) [34]	In vitro study
Khan et al. (2020) [2]	Viral load or Ct value changes was not reported
Jain et al. (2021) [35]	In vitro study
Huang et al. (2021) [36]	Viral load or Ct value changes was not reported
Carroul et al. (2020) [12]	Technical notes
Filho et al. (2021) [37]	Technical notes
Almanza-Reyes et al. (2021) [38]	Viral load or Ct value changes of participants was not reported

### Assessment of methodological quality

As shown in Table 2, according to the modified D&B score, 5 studies obtained a score of 6 [23, 24, 26–28], 1 study scored 7 [31], and 3 studies scored 8 [25, 29, 30]. All studies were considered to have a low risk of bias.

There was hetreogenecity in included studies in the type mouthwash (intervention), diagnostic kit, specimen (saliva, nasopharynx, or oropharynx swab) and time of experiment (the time between the first RT-PCR test and using the mouthwash). GRADE system ranked the certainty of the scientific evidence and the strength of the recommendation as moderate for both outcomes (Downgraded for observed heterogeneity) [22].

### Study characteristics

In 7 publications, the study population was patients with a positive PCR test for SARS-COV-2 in the hospital [23–25, 27–30]. In one study, the patients were quarantined at home or were admitted to the hospital [26]. The patients were those referred to Dental Clinics of The Ohio State University College of Dentistry and Wexner Medical Center in one study [31].

Six studies had control groups [24, 25, 28–31]. The other three studies had no control groups and baseline samples were compared with experimental samples [23, 26, 27].

In one study, if patients started one treatment for Covid-19, they were excluded from the study [24]. In two studies, the patients received different treatments for Covid-19 during the experiment such as lopinavir/ritonavir, hydroxychloroquine, antibiotics, or a combination of them [25, 27]. Seven studies did not the use of antiviral or other medications during the study [23, 24, 26, 28–31].

In one study, 9 out of 10 patients had different underlying diseases such as chronic renal failure, multiple myeloma, and arterial hypertension [23]. In another study, 2 out of 20 subjects had asthma and obesity as comorbidities [24]. A history of non-Hodgkin's lymphoma, diabetes, and ischemic stroke was reported for 2 out of 4 participants in one study [26]. About 30% of all patients had comorbidities in one study [28]. One study reported that a number of symptomatic patients received remdesivir or convalescent plasma but a number or percentage was not mentioned [31]. Another study reported that 21% of the participants had different underlying diseases (diabetes millets, hypertension, and chronic kidney disease) [25]. At least 30% of the participants had hypertension, cardiovascular disease, diabetes, respiratory disease, renal disease, obesity or hypothyroidism in one study [30] In another study, 77.84% of patients had no medical history [29]. One study did not mention any underlying diseases [27].

### Descriptive findings of studies

Studies conducted by Gottsauner et al. [23], Mohamed et al. [38], Mukhtar et al. [25], Carrouel et al. [29] and Chaudhary et al. [31] were not included in the meta-analysis. In the study by Gottsauner et al. [23], the envelope (E) gene of SARS-COV-2 was amplified. Four patients showed an increase in the viral load after intervention and 4 patients showed a decrease in the viral load. There was no difference in the viral load between baseline and intervention swab tests in two patients. Therefore, they reported no significant reduction in the intraoral viral load after rinsing with 1% hydrogen peroxide mouthwash (Tables 4, and  5). Mohamed et al. [24] reported the result of swab tests as either negative (no Ct obtained), positive (Ct value ≤ 45 for both assays), or indeterminate(When only one gene assay had Ct < 45) for E gene and RNA-dependent RNA polymerase (RdRp) gene before and after rinsing with PVP-I, Cetylpyridinium chloride (CPC), and tap water. SARS-CoV-2 test was negative in all specimens of PVP-I group on days 4, 6, and 12. In the Listerine group, 4 out of 5 swab tests were negative on subsequent days. Two samples were negative in the tap water group on days 4, 6 and 12. In the control group, one swab sample was negative on days 4 and 12, and there was no negative sample on day 6. In this study, rinsing with 1% PVP-I and Listerine mouthwashes three times a day effectively reduced the SARS-CoV-2 viral load. Writers concluded that rinsing 1% PVP-I and essential oils could be a part of the treatment and management of COVID-19 at early stages (Tables 4, and 5). Mukhtar et al. [25] reported the result of swab tests as either negative and inconclusive (Ct value = 35–40) or positive (Ct value < 34.99) for ORF-1a/b and E-genes after rinsing with a mouthwash containing 6% hydrogen peroxide (HP) mixed with 0.2% chlorhexidine gluconate (CHX). At baseline, Ct values of none of the swab tests were negative in the intervention and control groups (0 out of 46 swab test was negative). After 5 days, 6 out of 45 swab tests were negative in the intervention group while no swab test was negative in the control group. After 15 days, 15 out of 43 swab tests were negative in the intervention group and 9 out of 44 were negative in the control group. They found a significant difference in the PCR results between the two groups that used the mouthwash on day 5, but the difference was not significant on day 15. They concluded that their intervention caused more COVID19-negative PCR by 5 day of treatment, symptoms severity would be improved after 2 days and there would be less intubation and mortality (Tables 4, and 5). Carrouel et al. [29] targeted the RdRp gene. On days 1 and 7 of the experiment, the Ct value changed by 2 points at 1 h, 4 h, and 9 h after using the CDCM mouthwash, indicating that it was effective in reducing the viral load. According to the study by Keyarts et al. a 2-point increase in the Ct value was considered as effective in reducing the viral load [39]. Writers concluded that using CDCM on day 1 reduced the viral load of SARS-COV-2 (Tables 4, and 5). Chaudhary et al. [31] did not mentioned which RNA gene was targeted. Saline, 1% hydrogen peroxide, 0.12% chlorhexidine, and 0.5% povidone-iodine were effective 15 min and 45 min post mouthwash use according to Ct value reports. They concluded that mouthwashes can simply and effectively reduce the risk of transmitting the virus. Other characteristics and results of the 9 included studies are summarized in Tables 4 and  5.

**Table 4:** Characteristics of included studies

First author	Eligibility criteria	Patients’ characteristics	Lab. Test
Gottsauner [23]	Inclusion criteria: Positive covid19 infected patients within the last 72 h in a hospital Exclusion criteria: Patients who need intubation or mechanical ventilation or severe stomatitis	1 to 5 days (median 3 days) 12 patients (6 female and 6 male) had a median age of 55 years (range: 22–81 years). Two with neg. RT-PCR test	RT-PCR test of oropharyngeal specimens
Mohamed [24]	Inclusion criteria: Adults older than 18 years, COVID-19 positive patients with no symptom, less than five days from diagnosis Exclusion criteria: Objects who cannot understand instructions, express symptoms of covid-19 infection such as fever or respiratory problems or, abnormal chest computed tomography, patients started treatments for covid-19, objects infected with SARS-CoV-2 again, thyroid dysfunction, allergy to povidone-iodine	Age range from 22–56 years old (16 male,4 female)	RT-PCR test was performed on nasopharyngeal and oropharyngeal swabs targeting the E gene and RNA-dependent RNA polymerase gene (RdRP) and provided with a cycle threshold (ct) value
Mukhtar [25]	Inclusion criteria: Patients with positive PCR test for covid-19 through combined Nasopharyngeal Oropharyngeal swab who were hospitalized within 24 h Exclusion criteria: Objects under 18 years of age, mental or cognitive problems, pregnant women, head and neck injuries, patients who need intubation	The mean age was 49; the age range had no significant difference between the objects (P = 0.89). Number of males were higher (72 vs. 10) Intervention group (n = 46): 1 non-COVID Pneumonia (NCP) Asymptomatic; 11 NCP mild symptoms; 24 MILD COVID Pneumonia (CP); 1 moderate CP; 9 severe CP Control group (*n* = 46): 4 NCP Asymptomatic; 10 NCP mild symptoms; 18 mild CP; 3 moderate CP; 11 severe CP	RT-PCR test of nasopharyngeal and oropharyngeal swabs targeting the S, N and E-genes Obtaining CT value > 30 in subsequent covid-19 RT-PCR test
Lamas [26]	Inclusion criteria: Not mentioned Exclusion criteria: Not mentioned	74, 73, 43, 54 years old patients (28–41 days after positive nasopharyngeal positive test); 2 Males and 2 females	rRT-PCR assay which targeted E-gene, RdRP and N genes
Yoon [27]	Inclusion criteria: Not mentioned Exclusion criteria: Not mentioned	Two hospitalized patients diagnosed with covid-19	rRT-PCR which targeted the E and RdRP genes of SARS-CoV-2 Cts were derived from supplementary tables
Seneviratne [28]	Inclusion criteria: Patients whos their nasal swabs were positive for rRt-PCR assay of SARS-COV-2 from a hospital in Singapore Exclusion criteria: thyroid problems, patients received radioactive iodine lately, under treatment with lithium, pregnant women, and renal failure 19 pts had negative PCR for saliva and one patient excluded due to non-compliance	All were males except 1 in control group	The in-house RT-PCR test of saliva samples targeting the E gene of SARS-CoV-2 [Fold changes in comparison to control group also are reported in the article but we omit them as not reported in other studies.]
Carrouel [29]	Inclusion criteria: Adults aged 18to 85 years old with a clinical diagnosis of COVID-19 infection, asymptomatic or mild clinical symptoms that had been present for < 8 days Exclusion criteria: Pregnancy, breastfeeding, an inability to comply with the protocol, a lack of written agreement, mouthwash use on a regular basis (more than once a week), an inability to answer questions and a lack of cooperation	who had voluntarily presented at the hospital for a screening qualitative PCR test. Asymptomatic patients are defined as individuals without clinical signs whereas mild corresponds to outpatients and patients with clinical symptoms without pneumonia manifestations on image results	Quantitative RT-PCR (Data are expressed in log10 copies/mL of saliva or in % for the % of variation calculated with values expressed in log10 copies/mL.)
Eduardo [30]	Inclusion criteria: Age of 18–90 years - Length of hospitalization up to 3 days - Previously identified to be positive for SARS-CoV-2 as determined by nasal swabbing and qRT-PCR - Adequate performance in the use of the different types of mouthwash - Adequate performance of oral hygiene Exclusion criteria: No detection of SARS-CoV-2 by qRT-PCR at the time of recruitment - Hospitalized in intensive care units - Lesions in the oral mucosa - Bleeding in the oral cavity that prevented the collection of samples - History of allergy, irritations, or other side effects of the use of the test substances - Use of the test substances or other oral antimicrobials 48 h before the baseline collection - Non-adherence to the established protocol or inability to perform planned procedures	Patients hospitalized in negative-pressure rooms at the Hospital Israelita Albert Einstein (HIAE), Brazil, between June 2020 and July 2020,	Amplification of the SARS-CoV-2 N and ORF1ab genes was performed using a commercial COVID-19 qRT-PCR kit
Chaudhary [31]	Inclusion criteria: Adults age 21–80 admitted to The Ohio State University Wexner Medical Center with a diagnosis of COVID-19 confirmed by Polymerase Chain Reaction (PCR) for symptomatic group and (2) absence of any COVID-19 screening symptoms (based on the ADA questionnaire and body temperature) for the asymptomatic, pre-symptomatic and post-symptomatic groups Exclusion criteria: (1) Allergy to any study mouth rinse, (2) active uncontrolled thyroid disease, (3) pregnancy and (4) patients undergoing radioactive iodine therapy	Patients categorized to 4 groups: Asymptomatic, presymptomatic, post symptomatic	SARS-COV-2 N1, N2 genes were targeted

**Table 5:** Characteristics and results of included studies

First author	Type of study	No. of participants	Kind of mouthwash	Treatment schedule	Specimen	Testing time after intervention	Sample size	Baseline specimens analysis	Interventional specimens analysis
Gottsauner [23]	Clinical Pilot Study	10	Hydrogen peroxide 1%	Gargle for 30 s	Oropharyngeal specimens	30 min	5	Positive: 1 Negative: 4 (culture)	Positive: 0 Negative: 5 (culture)
Mohamed [24]	4-arms preliminary interventional study	20	1% PVP-I	gargle for 30 s, three times per day for 7 days	Nasopharyngeal and oropharyngeal swab	4d	5	___	Positive: 0 Negative: 5
						6d	5	___	
						12d	5	___	
			Listerine (essential oil)	gargle for 30 s, three times per day for 7 days	Nasopharyngeal and oropharyngeal swab	4d	5	___	Positive: 1 Negative: 4
						6d	5	___	Positive: 1 Negative: 4
						12d	5	___	Positive: 0 Intermediate: 1 Negative: 4
			Tap water	gargle for 30 s, three times per day for 7 days	Nasopharyngeal and oropharyngeal swab	4d	5	___	Positive: 3 Negative: 2
						6d	5	___	Positive: 1 Intermediate: 2 Negative: 2
						12d	5	___	Positive: 2 Intermediate: 1 Negative: 2
			No intervention	___	Nasopharyngeal and oropharyngeal swab	4d	5	___	Positive: 2 Intermediate: 2 Negative: 1
						6d	5	___	Positive: 3 Intermediate: 2 Negative: 0
						12d	5	___	Positive: 3 Intermediate: 1 Negative: 1
Mukhtar [25]	an investigator-initiated, randomized, phase IV clinical trial	92	10 ml of 0.2% Chlorhexidine gluconate and 5 ml of 6% Hydrogen peroxide ( a final concentration of 2%)	Gargling 15 ml three times daily 30 s for 2w Initially, they were advised to use the mouthwash for one minute (not exceeding 2 min contact time with the oral cavity); however, due to the difficulty of prolonged use given the high oxygen requirements …	Nasopharyngeal and oropharyngeal swabs	5d	Baseline: 46 5d: 45	Positive: 46 Inconclusive: 0 Negative: 0 Mean: 22.6 [95% CI: 20.8–24.3]	Positive: 34 Inconclusive: 5 Negative: 6
						15d	Baseline: 46 15d: 43	Positive: 46 Inconclusive: 0 Negative: 0 Mean: 22.6 [95% CI: 20.8–24.3]	Positive: 14 Inconclusive: 14 Negative: 15
			Control	–-	Nasopharyngeal and oropharyngeal swabs	5d	Baseline: 46 5d: 44	Positive: 46 Inconclusive: 0 Negative: 0 Mean: 23.7 [95% CI: 21.9–25.5]	Positive: 38 Inconclusive: 6 Negative: 0
						15d	Baseline: 46 15d: 44	Positive:46 Inconclusive: 0 Negative: 0 Mean: 23.7 [95% CI: 21.9–25.5]	Positive: 18 Inconclusive: 17 Negative: 9
Lamas [26]	Quasi-experimental	4	1% povidone iodine	15 ml for 1 min	Nasopharyngeal	–-	4	Positive:2 Negative: 2 Ct E 27.83 ± 11.33 Ct RdRp 29.94 ± 11.27 Ct N 30.12 ± 9.82	
					Saliva	5 min	4	Ct E 28.98 ± 7.59 Ct RdRp 32.39 ± 5.25 Ct N 32.70 ± 5.20	Ct E 29.35 ± 4.35 Ct RdRp 32.07 ± 2.64 Ct N 32.41 ± 3.85
					Saliva	1 h	4	Ct E 28.98 ± 7.59 Ct RdRp 32.39 ± 5.25 Ct N 32.70 ± 5.20	Ct E 33.62 ± 2.30 Ct RdRp 37.08 ± 0.59 Ct N 36.06 ± 1.32
					Saliva	2 h	4	Ct E 28.98 ± 7.59 Ct RdRp 32.39 ± 5.25 Ct N 32.70 ± 5.20	Ct E 35.88 ± 1.95 Ct RdRp 38.45 ± 0.60 Ct N 37.46 ± 2.43
					Saliva	3 h	4	Ct E 28.98 ± 7.59 Ct RdRp 32.39 ± 5.25 Ct N 32.70 ± 5.20	Ct E 35.38 ± 3.59 Ct RdRp 35.32 ± 2.91 Ct N 36.62 ± 1.78
Yoon [27]	Quasi-experimental	2	CHX 0.12%	15 ml, 30 sex, Gargling	Nasopharynx	Day1	2	19.38 ± 2.56	
						Day3	2	24.21 ± 0.53	
						Day5	2	25.07 ± 4.33	
						Day7	2	23.17 ± 6.93	
						Day9	2	36.12 ± 2.41	
					Oropharynx	Day1	2	25.75 ± 1.82	
						Day3	2	35.29 ± 3.04	
						Day5	2	30.51 ± 1.25	
						Day7	2	0	
						Day9	2	0	
					Saliva	Day1	2	23.61 ± 1.27	
						Day3	2	27.52 ± 5.49	
						Day5	2	30.69 ± 0.59	
						Day6	2	32.13 ± 1.77	
						Day7	2	0	
						Day9	2	39.67 ± 0.21	
					Saliva-Day 3 of hospitalization (day 6 of disease)	1 h	2	27.52 ± 5.49	0 (not detected)
						2 h	2	27.52 ± 5.49	0 (not detected)
						4 h	2	27.52 ± 5.49	30.16 ± 6.57
					Saliva-Day 6 of hospitalization (day 9 of disease)	1 h	2	32.13 ± 1.77	33.55 ± 2.13
						2 h	2	32.13 ± 1.77	37.17 ± 2.52
						4 h	2	32.13 ± 1.77	32.85 ± 9.75
Seneviratne [28]	Randomized clinical trial	16	–-		Saliva	Baseline	16	Positive: 17 Negative: 19 27.7 ± 4.8 (n = 16)	
			Povidone iodine (PI)	5 ml, 0.5% w/v	Saliva	5 min	4	22.53 ± 5.42	24.20 ± 8.08
						3 h	4	22.53 ± 5.42	24.21 ± 5.63
						6 h	4	22.53 ± 5.42	23.03 ± 5.17
			CHX	15 ml, 0.2% w/v	Saliva	5 min	6	29.90 ± 2.41	27.89 ± 2.57
						3 h	6	29.90 ± 2.41	30.01 ± 1.82
						6 h	6	29.90 ± 2.41	27.90 ± 2.34
			CPC	20 ml, 0.075%	Saliva	5 min	4	32.08 ± 2.27	32.91 ± 2.48
						3 h	4	32.08 ± 2.27	30.65 ± 3.20
						6 h	4	32.08 ± 2.27	31.86 ± 2.76
			Water (control)	15 ml	Saliva	5 min	2	26.33 ± 1.83	25.30 ± 2.17
						3 h	2	26.33 ± 1.83	23.16 ± 1.13
						6 h	2	26.33 ± 1.83	22.00 ± 2.80
Carrouel [29]	double-blind randomized controlled trial with two parallel arms	176	b-cyclodextrin (0.1%) and citrox (0.01%) (CDCM)	Participants were instructed to use three mouthwashes per day (at 09.00, 14.00 and 19.00), with either 30 mL of CDCM or placebo for 1 min	Saliva	Baseline (T1: day 1, 9:00am)	88	log10 copies/mL Median (IQR) 4.05 (2.94–4.96) mean: 3.87 SD: 1.25 Ct value: 30.09 SD:4,135	
						4 h (T2: day 1, 13:00)	88	log10 copies/mL Median (IQR) 4.05 (2.94–4.96) mean: 3.87 SD: 1.25 Ct value: 30.09 SD:4,135	log10 copies/mL Median (IQR) 3.33 (2.29–4.23) mean: 3.19 SD**:1.18** Ct value: 32.34 SD:3.90 Median difference T1-T2 (IQR) -0.38 (-1.39 to 0.00)
									% decrease T1-T2 median (IQR) -12.58% (-29.55% to -0.16%)
						9 h (T3: day 1, 18:00)	88	log10 copies/mL Median (IQR) 4.05 (2.94–4.96) mean: 3.87 SD: 1.25 Ct value: 30.09 SD:4,135	log10 copies/mL Median (IQR) 3.08 (0–4.19) Mean: 2.88 SD: 0.91 Ct value: 33.37 SD:3.01 Median difference T1-T3 (IQR) -0.24 (-1.55 to 0.06)
									% decrease T1-T3 median (IQR) -10.67% (-37.30% to 3.25%)
						Day 7	88	log10 copies/mL Median (IQR) 4.05 (2.94–4.96) mean: 3.87 SD: 1.25 Ct value: 30.09 SD:4,135	log10 copies/mL Median (IQR) 0 (0–1.34) Meadn: 0.78 SD:0.6 Ct: 40.31 SD: 1.98 Median difference T1-day 7 (IQR) -2.07 (-4.03 to -0.50)
									% decrease T1-day 7 median (IQR) -58.62% (-100% to -34.36%)
			Placebo	Placebo	Saliva	Baseline (T1: day 1, 9:00am)	88	log10 copies/mL Median (IQR) 3.85 (2.97–5.08) Mean: 4.01 SD: 0.87	
						4 h (T2: day 1, 13:00)	88	log10 copies/mL Median (IQR) 3.85 (2.97–5.08) Mean: 4.01 SD: 0.87 Ct value: 29.63 SD:2.87	log10 copies/mL Median (IQR) 3.60 (2.07–4.83) mean: 3.46 SD: 1.34 Ct value: 30.09 SD:4.43 Median difference T1-T2 (IQR) -0.15 (-0.97 to 0.33)
									% decrease T1-T2 median (IQR) -6.74% (-21.16% to 10.44%)
						9 h (T3: day 1, 18:00)	88	log10 copies/mL Median (IQR) 3.85 (2.97–5.08) Mean: 4.01 SD: 0.87 Ct value: 29.63 SD:2.87	log10 copies/mL Median (IQR) 3.31 (1.18–4.75) mean: 3.17 SD: 1.20 CT value: 32.41 SD: 3.96 Median difference T1-T3 (IQR) -0.30 (-1.23 to 0.22)
									% decrease T1-T3 median (IQR) -9.79% (e28.53% to 9.21%)
						Day 7	88	log10 copies/mL Median (IQR) 3.85 (2.97–5.08) Mean: 4.01 SD: 0.87 Ct value: 29.63 SD:2.87	log10 copies/mL Median (IQR) 1.62 (0–1.70) Mean: 1.38 SD:0.32 Ct value: 38.33 SD:1.05 Median difference T1-day 7 (IQR) -2.11 (-3.35 to -0.86)
									% decrease T1-day 7 median (IQR) -50.62% (-100% to -27.66%)
Eduardo [30]	randomized, double-blinded, placebo-controlled, single-center pilot clinical trial	60	Placebo	rinse with 20 mL for 1 min	Saliva	Immediately after rinsing	9	Positive: 100% Negative: 0% 30.07 ± 1.92	Positive: 100% Negative: 0% 29.46 ± 3.16
						30 min	9	Positive: 100% Negative: 0% 30.07 ± 1.92	Positive: 100% Negative: 0% 28.85 ± 2.90
						60 min	9	Positive: 100% Negative: 0% 30.07 ± 1.92	Positive: 100% Negative: 0% 29.03 ± 2.92
			CPC + Zn (0.075% cetylpyridinium chloride plus 0.28% zinc lactate (CPC ‏ Zn)	rinse with 20 mL for 30 s	Saliva	Immediately after rinsing	7	Positive: 100% Negative: 0% 28.16 ± 3.53	Positive: 100% Negative: 0% 31.89 ± 4.46
						30 min	7	Positive: 100% Negative: 0% 28.16 ± 3.53	Positive: 100% Negative: 0% 29.07 ± 5.54
						60 min	7	Positive: 100% Negative: 0% 28.16 ± 3.53	Positive: 100% Negative: 0% 28.66 ± 6.52
			HP (1.5% hydrogen peroxide)	rinse with 10 mL for 1 min	Saliva	Immediately after rinsing	7	Positive: 100% Negative: 0% 28.73 ± 3.40	Positive: 100% Negative: 0% 32.03 ± 5.19
						30 min	7	Positive: 100% Negative: 0% 28.73 ± 3.40	Positive: 100% Negative: 0% 30.15 ± 3.93
						60 min	7	Positive: 100% Negative: 0% 28.73 ± 3.40	Positive: 100% Negative: 0% 24.54 ± 6.06
			CHX (0.12% chlorhexidine gluconate)	rinse with 15 mL for 30 s	Saliva	Immediately after rinsing	8	Positive: 100% Negative: 0% 26.35 ± 6.20	Positive: 100% Negative: 0% 26.78 ± 5.76
						30 min	8	Positive: 100% Negative: 0% 26.35 ± 6.20	Positive: 100% Negative: 0% 27.77 ± 5.95
						60 min	8	Positive: 100% Negative: 0% 26.35 ± 6.20	Positive: 100% Negative: 0% 27.46 ± 5.59
			HP + CHX	rinse with 10 mL of HP mouthwash for 1 min, followed by rinsing with 15 mL of CHX mouthwash for 30 s	Saliva	Immediately after rinsing	12	Positive: 100% Negative: 0% 30.74 ± 5.50	Positive: 100% Negative: 0% 31.20 ± 6.74
						30 min	12	Positive: 100% Negative: 0% 30.74 ± 5.50	Positive: 100% Negative: 0% 30.24 ± 7.24
						60 min	12	Positive: 100% Negative: 0% 30.74 ± 5.50	Positive: 100% Negative: 0% 28.35 ± 8.68
Chaudhary [31]	Randomized, triple-blinded study	40 out of 200	1% hydrogen peroxide, 0.12% chlorhexidine gluconate or 0.5% povidone-iodine	7.5 ml of the mouth rinse for 30 s	Saliva	15 and 45 min post-rinsing	40 subjects in each group	–-	All four mouth rinses reduced salivary carriage of SARS-CoV-2. A median reduction of 61–89% (mean of 25–74%) was observed at 15 min, while the median reduction ranged from 70–97% at 45 min (mean of 30–43%). Neither the 15-miniute reduction in viral load, nor the persistence of reduction at 45 min differed between the mouthrinses (*p* > 0.05, Dunn’s test)

### Meta-analysis

**Fig. 2:**
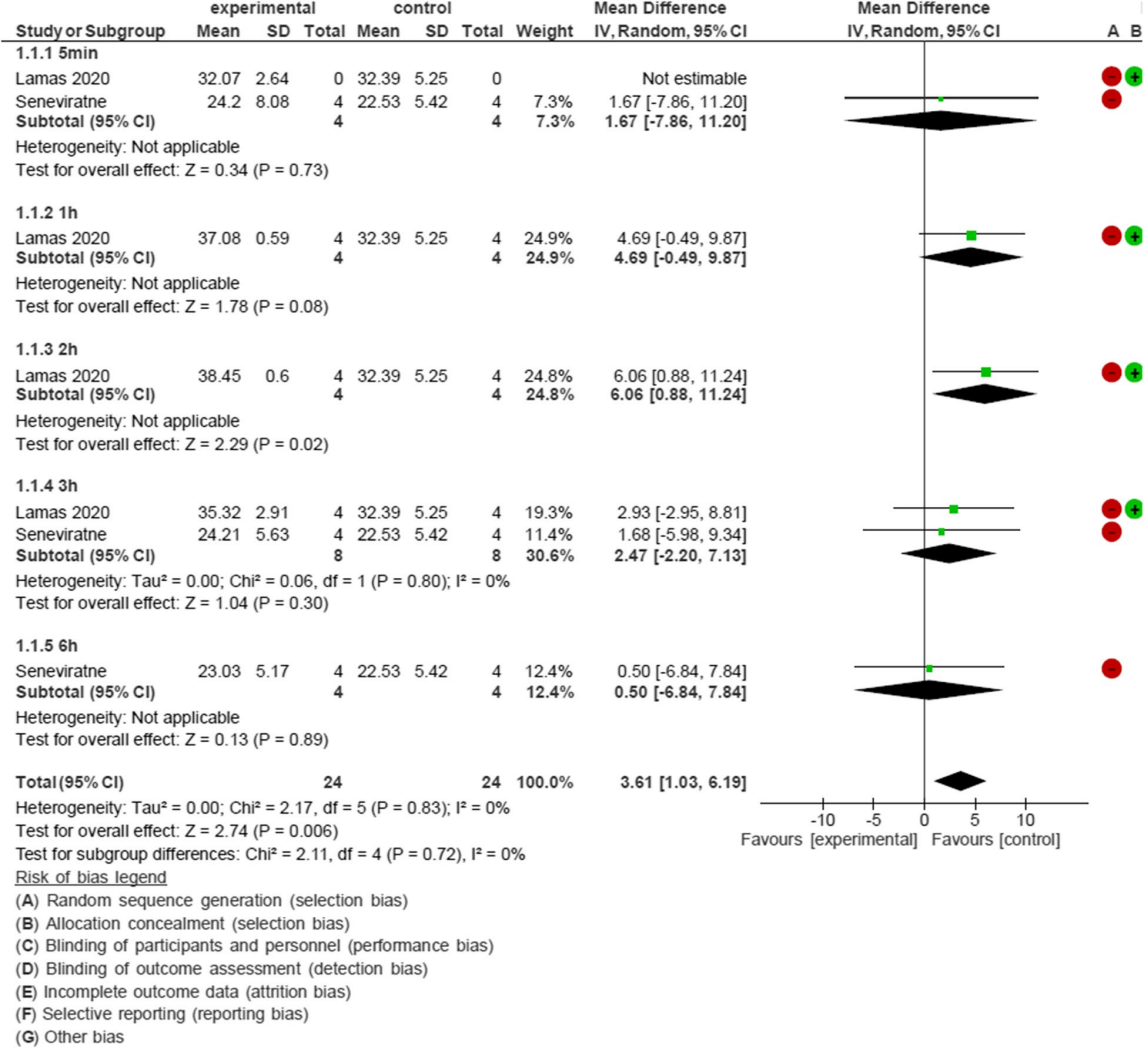
Forest plot of meta-analysis of the effect of PVP-I mouthwash on Cycle threshold value of SARS-COV-2

According to the mean differences of Ct values, 4 studies were included in meta-analysis [26–28, 30]. These studies used mouthwashes containing PVP-I, CHX and CPC. In this meta-analysis, there were 5 subgroups of time for the PVP-I-containing mouthwash: 5 min (min), 1 h (h), 2 h, 3 h, and 6 h after rinsing. The studies conducted by Lamas and Seneviratne [26, 28] were included in the meta-analysis of the effect of PVP-I-containing mouthwash on the Ct value of SARS-COV-2. The MD was 3.61 and 95% confidence interval (CI) was 1.03 to 6.19 for analyzing Ct values before and after rinsing with PVP-I containing mouthwashes. These mouthwashes were found to be effective 5 min, 1 h, 2 h, 3 h and 6 h after rinsing (Fig. 2).

**Fig. 3:**
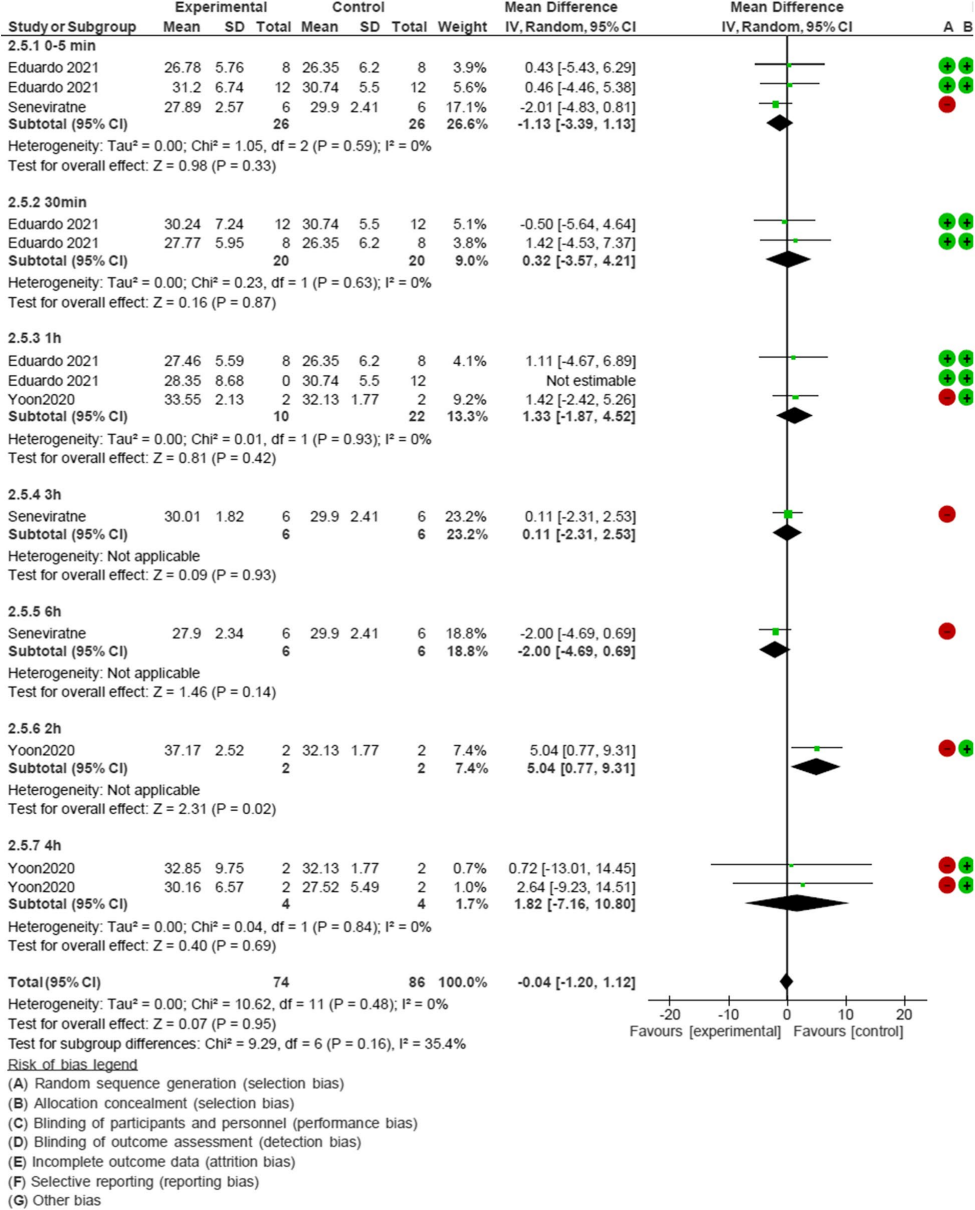
Forest plot of meta-analysis of the effect of Chlorhexidine gluconate-containing mouthwash on Cycle threshold value of SARS-COV-2

The meta-analysis of the effect of CHX mouthwash on Ct value of SARS-COV-2, which included studies conducted by Yoon, Seneviratne and Eduardo [27, 28, 30], had 7 time subgroups: 0–5 min, 30 min, 1 h, 2 h, 3 h, 4 h and 6 h after rinsing. MD was -0.04 and 95% CI was -1.20 to 1.12 for analyzing Ct values before and after rinsing with mouthwashes containing CHX; therefore, these mouthrinses were not effective for reducing SARS-COV-2 viral load (Fig. 3).

**Fig. 4:**
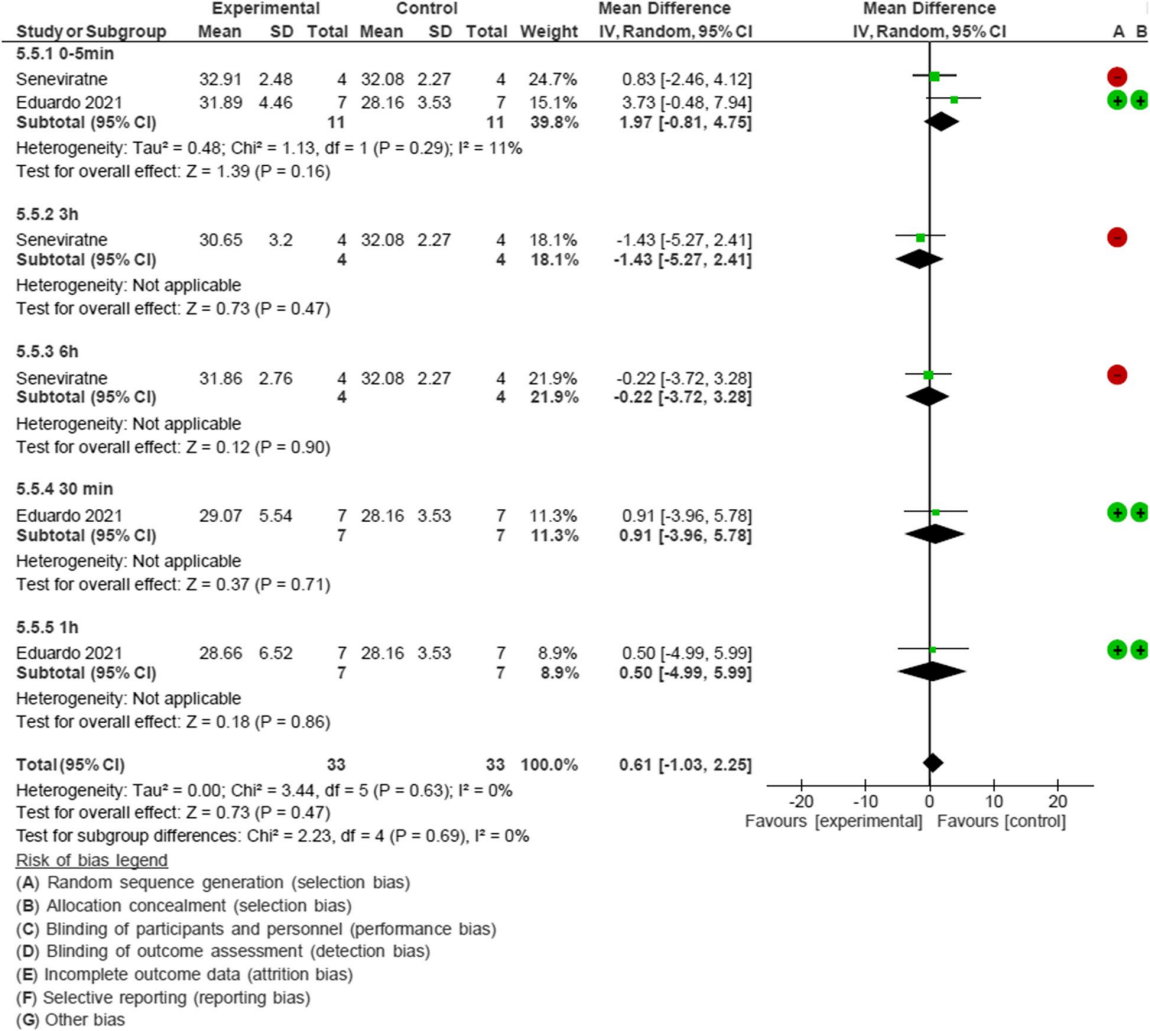
Forest plot of meta-analysis of the effect of CPC-containing mouthwash on Cycle threshold value of SARS-COV-2

There were five subgroups of time in the meta-analysis of CPC-containing mouthwashes: 0–5 min, 30 min, 1 h, 3 h, 6 h after rinsing according to the studies conducted by Seneviratne and Eduardo [28, 30]. CPC containing mouthwashes were not effective against SARS-COV-2 when analyzing Ct values before and after rinsing mouthwashes containing CPC (MD: 0.61, 95% CI: -1.03 to 2.25) (Fig. 4).

**Fig. 5:**
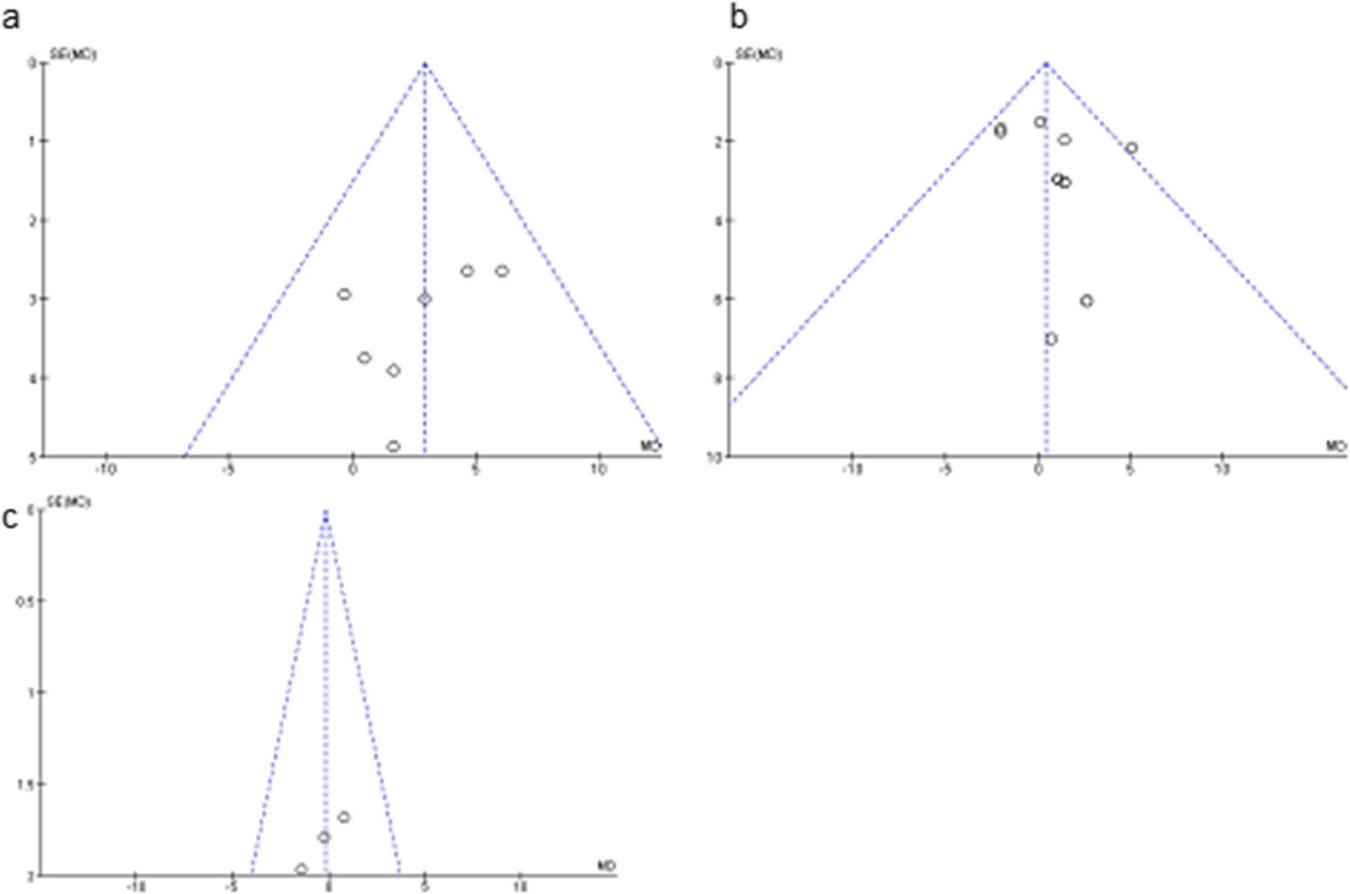
Funnel plots based on Ct value changes. **a**) PVP mouthwash **b**) CHX mouthwash **C**) CPC mouthwash

The Egger’s and Begg’s tests were used for publication bias assessment (Fig. 5). Although a specific gap cannot be detected, due to the small number of included studies, the evaluation of publication bias is not reliable.

## Discussion

Covid-19 is known to transfer from one person to another through infected droplets and aerosols. Close contact of dentists with patients and aerosol-generating procedures can significantly increase airborne contamination and cross-infection of SARS-CoV-2 in dental clinics.

Antiseptic mouthrinses have been suggested for various prophylactic and therapeutic purposes in dentistry. However, their anti-SARS-CoV-2 effect to control the viral load has not been evaluated systematically.

Mouthwashes should have a high substantivity. It means that they are released slowly, so they show their antimicrobial effects for an extended time; therefore, only mouthrinses with high substantivity may be effective against Covid-19.

In-vitro studies demonstrated that different concentrations of povidone-iodine have antiviral effects against SARSCOV-2 [15–18]. Some other studies investigated the effects of hydrogen peroxide, cetylpyridinium chloride, ethanol, and essential oil mouthwashes on Covid-19 [15, 19]. An in-vitro study examined the virucidal effects of 8 different oral rinses. In this study, researchers added mouthrinses to viral suspension and a particular substance simulating the oral environment. The results showed that dequalinium chloride, benzalkonium chloride, ethanol, and povidone-iodine had significantly more antiviral effects compared to other compounds. They concluded that commercially available oral rinses inactivated SARS-CoV-2 within a short exposure time [15].

Hydrogen peroxide eliminates microorganisms of the oral cavity by degradation into oxygen and water. Hossainian et al. found that hydrogen peroxide mouthwashes did not consistently control the microbiota of the oral cavity [40]. Despite the safety of hydrogen peroxide in the short time, long-term use might have carcinogenic effects. According to Filho J et al., H_2_O_2_ mouthwashes should not be continuously recommended for patients with Covid-19 because there is no approved evidence that H_2_O_2_ prevents Covid-19 syndromes or prevents the virus from spreading [41]. However, Peng et al. found that 1% hydrogen peroxide or 0.2% povidone-iodine reduced the microbial and viral load when using a rubber dam was not possible [8]. In the oral cavity, hydrogen peroxide will be inactivated due to the host catalase activity [42].

PVP-I is a water-soluble iodophor composed of iodine and polyvinylpyrrolidone as a water-soluble polymer [43]. The free iodine molecule penetrates the microorganism, oxidizes surface proteins, and disrupts nucleotides and fatty acids, causing cell death [43]. Povidone‐iodine has a broad spectrum of antimicrobial effects against bacteria, fungi and different viruses. In one study, 0.23% povidone-iodine mouthrinse showed a significant reduction in bactericidal activity and inactivated influenza virus and MERS-COV [16]. PVP-I is more effective than other common antimicrobial agents such as chlorhexidine, Octenidine, and polyhexinide [44]. It has been demonstrated that PVP-I had sustained effects for more than 4 h [45]. Oxidation mouthwashes, such as povidone-iodine may reduce the salivary viral load of SARS-COV-2 [46]. A study by Muhamed Khan et al. confirmed that gargling a mouthwash containing 0.5% povidone-iodine was safe for healthcare workers and their patients before oral surgery and ENT examination. No allergy was reported [2]. Parhar et al. found that PVP-I reduced the viral transmission of Covid-19 during upper airway mucosal surgery [47]. There are some contraindication to the use for PVP-I: 1) patients with an allergy to iodine, 2) thyroid disease, 3) pregnancy, 4) treatment with radioactive iodine [48]. Our meta-analysis showed that PVP-I mouthwash could reduce the viral load of SARS-COV-2 in the oral cavity.

CHX is a broad-spectrum antiseptic mouthwash with antibacterial and antiplaque properties [49, 50]. Bernstein et al. reported that CHX has antiviral effects on lipid-enveloped viruses while it has no effects on non-enveloped viruses [51]. In a systematic review by Cavalcante-Leão that included in-vitro studies, the researchers concluded that the use of 1% and 7% PVP-I was more effective than HP and CHX in reducing the viral load of the coronavirus family [52]. Peng et al. also found that CHX was not effective for Covi-19 transmission reduction during dental practices [8]. According to the results of the meta-analysis, it may not be concluded whether CHX or a combination of CHX and HP has antiviral effects against SARS-CoV-2.

Listerine mouthrinses contain four active ingredients (eucalyptol, menthol, methyl salicylate, thymol) as well as inactive constituents such as water, alcohol and benzoic acid. Previous studies demonstrated the effectiveness of Listerine in reducing dental plaque and gingivitis [53]. Moreover, Listerine has a significant efficacy against fungal species. Listerine disrupts the cell walls of microorganisms and inhibits the enzymatic activity of pathogens [54]. In vitro studies have shown that Listerine has virucidal effects. Meiller et al. found that oral rinsing with Listerine for thirty seconds reduced the viral load of HSV-1. They explained that this finding could be extended to other enveloped viruses [55]. Mohamed et al. concluded that Listerine mouthwash was effective against SARS-CoV-2 [38]; however, the study by Mohamed et al. [38] was used for the systematic review but it was not included in the meta-analysis.

CDCM mouthwash contains beta-cyclodextrin and Citrox. A study by Carrouel et al. that evaluated this compound was included in the present systematic review and meta-analysis. Hooper et al. found that 1% CDCM mouthwash significantly inhibited the growth of 14 bacterial and some candida species [56]. It is also effective against Zikavirus [57], enterovirus A71 [58], HIV-1 [59], and influenza A [60]. However, no other published study evaluated the effect of this component on SARS-COV-2 except for the study conducted by Carrouel et al. This study was used for the systematic review but it was not included in the meta-analysis.

CPC is a quaternary ammonium water-soluble compound. CPC can penetrate the cell membrane, raise the endocytic and lysosomal PH, and disrupt the cell activity. In past decades, some clinical trials showed that CPC mouthwashes were effective in gingivitis and plaque control [61]. Gurzawska-Comis et al. found that CPC might have virucidal effects, especially against enveloped viruses [62]. In-vitro studies suggest that CPC disrupts different strains of the influenza virus [63]. Using CPC-containing mouthwashes may not be effective in reducing Covid-19 viral load according to our meta-analysis.

A limited number of clinical trial studies examined the effect of mouthwashes on the viral load of Covid-19 in the saliva. The sample size of some of these experimental studies was small. Therefore, more clinical trial studies with standard sample sizes are required.

## Conclusion

Since the oral cavity serves as a reservoir of SARS-CoV-2, using mouthwashes can be effective in Covid-19 patients to prevent the transmission of this virus. PVP-I at 0.5% and 1% concentrations reduced the viral load of SARS-CoV-2 in oropharyngeal, nasopharyngeal, and saliva specimens. Thus, it might be considered as a simple and inexpensive intervention during the Covid-19 pandemic.

## Data Availability

All data generated or analysed during this study are included in this published article.

## Abbreviation

Ct: Cycle threshold
MD: Mean difference
PVP-I: Povidone-iodine
CPC: Cetylpyridinium chloride
CHX: Chlorhexidine gluconate
ACE2: Angiotensin-converting enzyme-2
RdRp: RNA-dependent RNA polymerase
PRISMA: Preferred Reporting Items for Systematic Reviews and Meta-Analyses
D&B checklist: Down and Black checklist
HP: Hydrogen peroxide
min: Minutes
h: Hour
CI: Confidence Interval
